# Supplementary material for: Smooth Interpolating Curves with Local Control and Monotone Alternating Curvature
Source: Comput Graph Forum. 2022 Oct 6;41(5):25–38. doi: 10.1111/cgf.14600 (PMC9827861; doi:10.1111/cgf.14600)
Supplement: Supplementary file 1 — Supplement Material [file CGF-41-25-s001.zip › Local-Smooth-Interpolating-MonoCurvature/extern/clothoids/docs/api-matlab/class_a00104.html]

Class Triangle2D — Clothoids v2.0.9

### Navigation

- index
- toc
- next
- previous
- Clothoids »
- MATLAB API »
- Class Triangle2D

# Class Triangle2D¶

- Defined in File Triangle2D.m

## Inheritance Relationships¶

### Base Type¶

- `public handle`

## Class Documentation¶

class Triangle2D : public handle¶
:   Public Functions

    function Triangle2D( varargin)¶
    :   Create a new C++ class instance for the triangle object

        **Usage:**

        ```
        ref = Triangle2D();
        ref = Triangle2D( x0, y0, x1, y1, x2, y2 );
        ref = Triangle2D( p0, p1, p2 );
        ```

        **On output:**

        - `ref`: reference handle to the object instance

    function obj\_handle( self)¶
    :   Destroy the C++ class instance.

    function is\_type( ignoredArg)¶

    function build( self, varargin)¶

    function translate( self, tx, ty)¶
    :   move the triangle by `(tx,ty)`

    function scale( self, sc)¶
    :   scale triangle by `sc` factor

    function points( self)¶

    function rotate( self, angle, cx, cy)¶
    :   Rotate the triangle by angle with center of rotation `(cx,cy)`

        **Usage:**

        ```
        ref.rotate(angle, cx, cy)
        ```

        **On input:**

        - `angle`: the angle of rotation
        - `cx`, `cy`: coordinates of the centre of rotation

    function isInside( self, x, y)¶

    function distanceMin( self, x, y)¶

    function distanceMax( self, x, y)¶

    function info( self)¶

    function overlap( self, obj)¶

    function plot( self, color, varargin)¶

    function plot2( self, varargin)¶

### Quick search

### Table of Contents

- Matlab Interface Manual
- C++ API
- MATLAB API

«
hide menu

menu
sidebar
»

### Navigation

- index
- toc
- next
- previous
- Clothoids »
- MATLAB API »
- Class Triangle2D

© Copyright 2021, Enrico Bertolazzi and Marco Frego.
Created using Sphinx 4.2.0.
